# Diagnosis of Brain Tumors Using Amino Acid Transport PET Imaging with ^18^F-fluciclovine: A Comparative Study with L-methyl-^11^C-methionine PET Imaging

**DOI:** 10.22038/aojnmb.2017.8843

**Published:** 2017

**Authors:** Naohiro Tsuyuguchi, Yuzo Terakawa, Takehiro Uda, Kosuke Nakajo, Yonehiro Kanemura

**Affiliations:** 1 Department of Neurosurgery, Osaka City University Graduate School of Medicine, Osaka, Japan; 2 Department of Neurosurgery, Asahikawa Medical University, Kansai Molecular Diagnosis Network for CNS Tumors, Osaka, Japan; 3 Department of Neurosurgery, Hokkaido Oono Memorial Hospital, Osaka, Japan; 4 Department of neurosyrgery, Kansai Molecular Diagnosis Network for CNS Tumors, Osaka National Hospital, Osaka, Japan

**Keywords:** ^11^C-Methionine, ^18^F-Fluciclovine, Glioma, Positron emission tomography

## Abstract

**Objective(s):**

^18^F-fluciclovine (trans-1-amino-3-[^18^F] fluorocyclobutanecarboxylic acid, [FACBC]) is an artificial amino acid radiotracer used for positron emission tomography (PET) studies, which is metabolically stable in vivo and has a long half-life. It has already been shown that FACBC-PET is useful for glioma imaging. However, there have been no reports evaluating the efficiency of FACBC-PET in the diagnosis of brain tumors in comparison with other PET tracers in clinical studies. The purpose of this study was to investigate the efficacy of FACBC-PET imaging in glioma diagnosis, compared to L-methyl-^^11^^C-methionine (MET)-PET.

**Methods:**

Six consecutive patients (four male, two female), who were clinically suspected of having high- or low-grade glioma, received both FACBC-PET and MET-PET within a two-week interval. T1-weighted, contrast-enhanced, T1-weighted, and fluid-attenuated inversion recovery magnetic resonance imaging was performed to assist with subsequent tissue resection. Visual findings and semi-quantitative analyses of FACBC and MET uptake, using standardized uptake values (SUVs) and lesion-to-contralateral normal brain tissue (LN) ratios, were evaluated to compare PET images.

**Results:**

SUVs for FACBC were lower than those for MET in the non-lesion cerebral cortex, brain stem, and cerebellar hemisphere. There was a weak positive correlation between FACBC and MET uptake in glioma tissue, although L/N ratios for FACBC were higher than those for MET in all the cases.

**Conclusion:**

FACBC-PET showed higher contrast than MET-PET by both visual and semi-quantitative analyses and may therefore provide better assessment for the detection of glioma. This study was registered as clinical trial (No. JapicCTI-132289).

## Introduction

Positron emission tomography (PET) imaging with amino acid analogs is often used for clinical applications, as it targets the increased amino acid transport of tumors and has proven more useful for detecting brain tumors than ^18^F-fludeoxyglucose PET. Methionine is an essential sulfur amino acid necessary for cell proliferation, and thus PET with L-methyl-^11^C-methionine (MET), which is mainly taken up by system L-amino acid transporters, is frequently used for brain tumor imaging (1, 2). For example, MET-PET has been successfully used to determine prognosis of malignant disease, assessing tumor extent, planning biopsy and radiotherapy, and differentiating between tumor recurrence and radiation necrosis in primary gliomas (3, 4).

High MET accumulation in primary brain tumors is considered to indicate malignant status and significantly unfavorable prognosis. Accordingly, MET-PET plays an important role in evaluating brain tumors, although it is difficult to evaluate benign tumors through this approach since they may show low MET uptake (5, 6).

Since MET-PET shows faint physiological uptake in normal brain (7), the boundary between tumor and non-diseased brain tissue can be ambiguous. There can also be strong physiological uptake in the nasal mucosa and bone marrow under certain inflammatory conditions. Finally, ^11^C-MET, which has an extremely short half-life (20 min), can only be used in limited institutions that are equipped with a cyclotron for its production.

At present, the artificial amino acid radiotracer ^18^F-fluciclovine (trans-1-amino-3-[^18^F]fluorocyclobutanecarboxylic acid, [FACBC]) is available for use in clinical PET studies (8, 9). FACBC labeled with ^18^F, which has a longer half-life (110 min) than MET, is useful for many departments with a PET scanner. However, there have been few reports on the efficacy of FACBC-PET for the diagnosis of brain tumors. Therefore, we aimed to compare the effectiveness of FACBC-PET and MET-PET imaging in the diagnosis of glioma.

## Methods

### Patient characteristics

From December 2013 to June 2014, six consecutive patients with suspected brain tumors (four male, two female) underwent both FACBC-PET and MET-PET within a two-week interval. Patients’ age ranged from 21 to 70 years (mean age: 44.2±18.5 years; Table 1). All the subjects were diagnosed with suspected glioma by magnetic resonance imaging (MRI). T1-weighted, contrast-enhanced, T1-weighted, and fluid-attenuated inversion recovery (FLAIR) were scheduled for brain tumor resection. This study was approved by the Institutional Review Board, Osaka City University, Graduate School of Medicine (approval numbers: #53 1994, and #3112 2015). All the enrolled patients received thorough explanations and displayed sufficient understanding prior to providing written informed consent.

**Table 1:** Case summary

Case	Age	Sex	Histological diagnosis	lesion	MIB-1	MGMT	1p/19q	IDH1	TP53	TERT	FACBC				MET			

											SUV	SUV	LN	LN	SUV	SUV	LN	LN

											mean	max	mean	max	mean	max	mean	max
1	40	F	Diffuse Astrocytoma	Lt Fr	3.5	+	-	+	+	-	1.61	2.74	3.85	6.54	2.91	4.40	2.02	3.06
2	61	M	Diffuse Astrocytoma	Lt T	3.7	+	-	+	+	-	0.55	1.07	1.76	3.40	1.46	2.56	1.42	2.48
3	21	M	Diffuse Astrocytoma	Rt In	4.9	+	-	+	+	-	0.69	1.12	1.76	2.84	1.21	1.79	1.08	1.60
4	57	M	Oligodendro-Glioma	Rt Fr	8.1	+	+	+	w	+	1.35	2.56	6.70	12.71	2.49	4.49	2.54	4.59
5	70	F	Anaplastic Astrocytoma	Lt Fr	5	+	-	-	w	+	1.54	2.36	3.35	5.12	2.22	3.51	1.88	2.97
6	43	M	Glioblastoma	Bil Fr	30	+	-	+	+	+	3.75	6.15	6.36	10.42	4.77	6.64	3.31	4.61

F: female, M: male, Rt: right, Lt: left, Bil: bilateral, Fr: frontal lobe, T: temporal lobe

In: insula, MGMT: MGMT methylation, 1 /19q: 1p/19q co-deletion, IDH1: IDH1 mutation, TP53: TP53 mutation, w: wild type, TERT: TERT mutation

### PET studies

FACBC-PET and MET-PET were performed in accordance with previously reported procedures (4, 7, 8). All the patients underwent MET- and FACBC-PET using the Biograph-16 scanner (lutetium oxyorthosilicate detector, LSO; Siemens, Bonn, Germany). FACBC was manufactured in accordance with Good Manufacturing Practices for investigational drugs and was supplied at a dose of 185 MBq per 2 ml (at the time of assay) by Nihon Medi-Physics Co., Ltd. (Tokyo, Japan) according to previously reported methods (8, 10).

Patients fasted for at least 4 h and then were treated with 2 ml of FACBC (mean dose of radioactivity: 235.5±35.2 MBq, range: 211.2–268.1 MBq) or MET (4 MBq/kg) by intravenous injection, followed by a flush with physiological saline. Emission scans of the brain were performed for a total of 10 min, after either 19 min of FACBC administration, or 20 min of MET administration. Axial and trans-axial PET resolutions in Biograph-16 were 5.5 and 5.9 mm (full width at half maximum), respectively. Emission scans were reconstructed to a matrix of 336×336 and attenuation and scatter correction were performed; voxel size was 1.02×1.02×2.00 mm.

### MRI imaging and tumor resection

Following PET scanning, additional MRI images (T1-weighted, contrast-enhanced, T1-weighted, and FLAIR) for the purpose of performing tissue resection were acquired using the Achieva 1.5 tesla imager (Philips Healthcare, Best, the Netherlands).

### Image analysis

**Figure 1:**
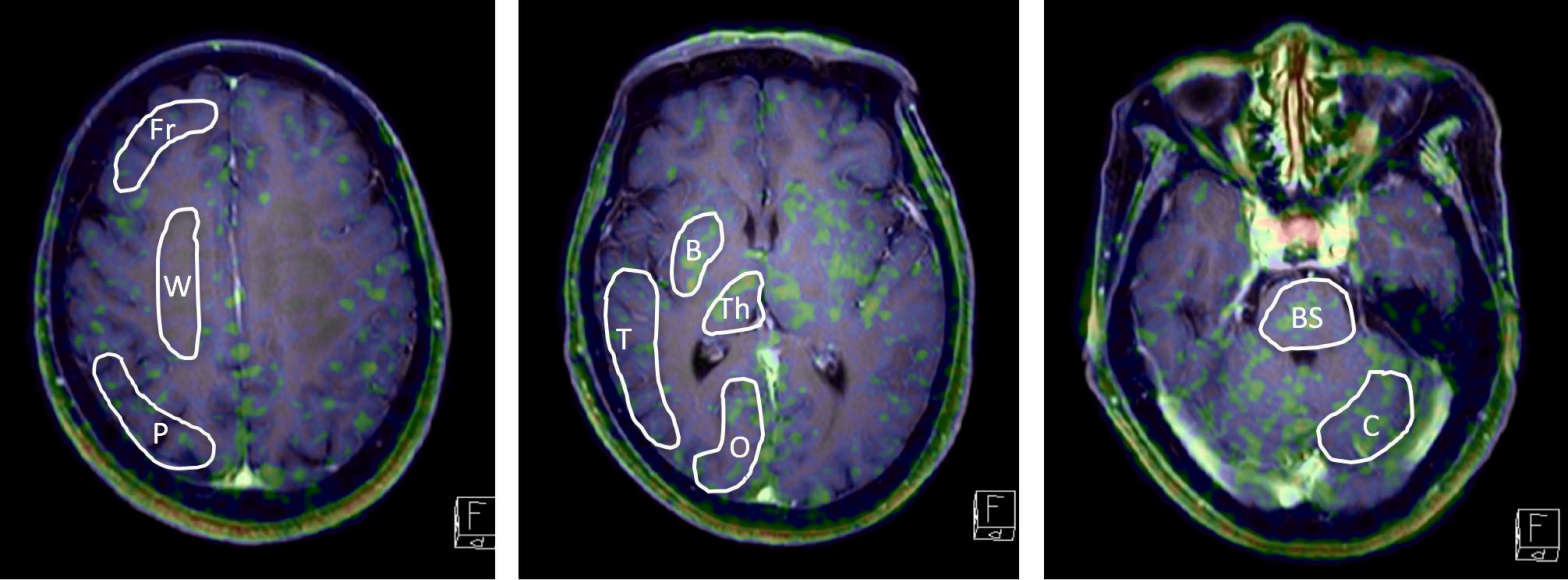
Manual ROIs in contralateral normal brain Fr: frontal lobe, P parietal lobe, T: temporal lobe, O: occipital lobe, B: basal ganglion, Th: thalamus, C: cerebellar cortex, Po: pons, W: white matter of corona radiata

PET scans were independently analyzed by two experts in the field of nuclear medicine and fused with those acquired by MRI using the Biograph-16 software for the navigation of each subject. PET data was analyzed using the same region of interest (ROI) settings as our previous study (4), manually placing irregular ROIs in the co-registered MRI image for each subject in the following locations: the lesion itself, the non-lesion side of the cerebral cortex (frontal, temporal, parietal, and occipital lobe), the non-lesion side of the cerebral white matter (the centrum semiovale), as well as the non-lesion side of the thalamus, basal ganglia, cerebellar hemisphere, and brain stem (Figure 1).

These ROIs were then transferred to the corresponding PET image to calculate the uptake of each radiotracer. Activity counts were normalized relative to the injected dose per kilogram of patient body weight (standardized uptake value, or SUV) as follows: SUV= (pixel count/pixel volume)/(injected radioisotope activity/body weight) × calibration factor. Mean and maximum SUVs (SUV_mean_ and SUV_max_, respectively) were calculated for semi-quantitative analysis of FACBC and MET uptake by each lesion. The mean and maximum lesion-to-contralateral normal brain tissue ratios (LN_mean_ and LN_max_) were determined by dividing the tumor SUV_mean_ and SUV_max_ by the SUV_mean_ of the contralateral cortex. The SUVs and L/N ratios were used as indicators of the degree of PET accumulation with all values being expressed as mean±SD.

### Pathological examination of the collected tissue specimens

The collected tissues were stained with hematoxylin and eosin, and immunohistochemistry was performed for glial fibrillary acidic protein (GFAP), 1p and 19q copy number status, telomerase reverse transcriptase (TERT) promoter, the methylation status of the O^6^-methylguanine DNA methyltransferase (MGMT) promoter, Ki-67 (MIB-1), and mutant isocitrate dehydrogenase 1 protein (IDH1 R132H). The genetic diagnosis of the resected tissues was made based on the 2016 World Health Organization (WHO) classification.

## Results

### Patients and lesions Characteristics

Histopathological diagnosis was confirmed by surgical resection. From the six patients, there were three diffuse astrocytomas, one oligodendroglioma, one anaplastic astrocytoma, and one glioblastoma (Table 1).

### Background uptake of FACBC

By visual examination of all the patients, there appeared to be lower uptake of FACBC than MET in normal brain tissue. FACBC was also found to accumulate in glandular tissue (pituitary gland, choroid plexus, and lacrimal gland) mucosa, and subcutaneously.

**Figure 2:**
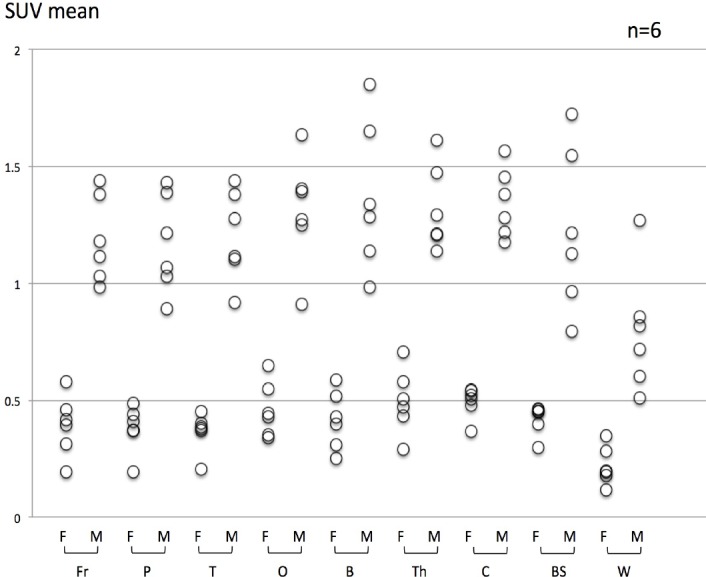
Comparison between FACBC and MET SUV_mean_ in each normal region SUV_mean_ of FACBC is lower than that of MET in each ROI. F: FACBC-PET, M: MET-PET Fr: frontal lobe, P parietal lobe, T: temporal lobe, O: occipital lobe, B: basal ganglion, Th: thalamus, C: cerebellar cortex, Po: pons, W: white matter of corona radiate

After calculating SUV_mean_ as a measure of tracer uptake, FACBC was confirmed to be lower than MET in all parts of the brain parenchyma as presented in Figure 2.

### Visual and semi-quantitative analysis of FACBC and MET uptake in glioma

**Figure 3:**
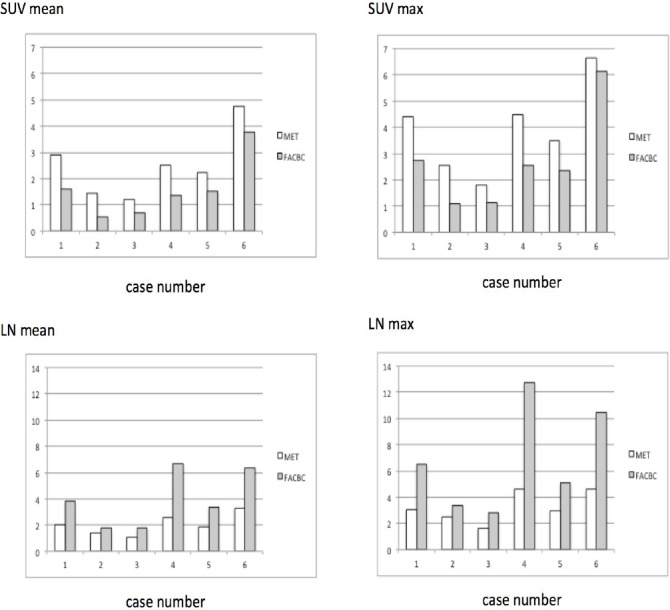
Comparison between FACBC and MET for SUV_mean_, SUV_max_, LN_mean_, and LN_max_ in the six cases. SUV_mean_ and SUV_max_ of FACBC are slightly lower than those of MET (Figures 3A and 3B) with no statistically significant differences. LN_mean_ and LN_max_ of FACBC are significantly higher than those of MET (Figure 3C and 3D).

FACBC and MET were found to show similar patterns of uptake in glioma (Figure 3), with low levels of uptake in low-grade gliomas (Figure 4; Cases 2 and 3) and higher levels of uptake in high-grade gliomas (Figure 4; Cases 5 and 6). In the oligodendroglioma case, which featured TERT mutation, IDH1 mutation, and 1p19q co-deletion, high levels of tracer uptake were found in both PET studies (Figure 4; Case 4). FACBC has low background accumulation, while image contrast was higher with FACBC-PET than MET-PET and the tumor was clearly depicted in cases 1, 4, 5, and 6. On the other hand, in low-grade gliomas (Cases 2 and 3), tumors showed low accumulation; thus, there was no visual difference between FACBC-PET and MET-PET scans. SUVs for MET in glioma were similar to SUVs for FACBC, but L/N_mean_ and L/N_max_ were higher in FACBC-PET than MET-PET (Figure 3).

**Figure 4. Case 1:**
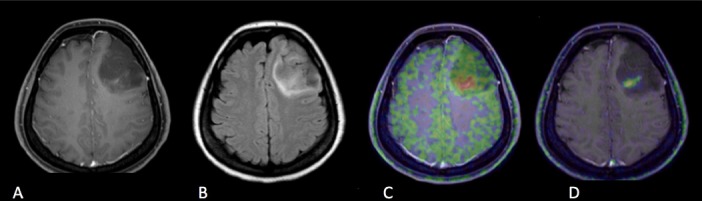
A: Gd contrast-enhanced T1-weighted image B: FLAIR (fluid-attenuated inversion recovery) image C: [^11^C]methionine positron emission tomography (MET-PET) D: anti- [^18^F]FACBC positron emission tomography (FACBC-PET) A 40-year-old lady who had poor enhancement homogenous shaped mass in the left frontal lobe in Gd T1-weighted image, which was diagnosed with diffuse astrocytoma, grade II, IDH mutant. FLAIR image showed the high signal lesion around the tumor which had no uptake in FACBC-PET and unclear uptake in MET-PET, and the lesion was diagnosed with edema. High uptake of MET was seen in small part of the tumor, while slight uptake of FACBC was detected against low uptake of background.

**Figure 4. Case 2:**
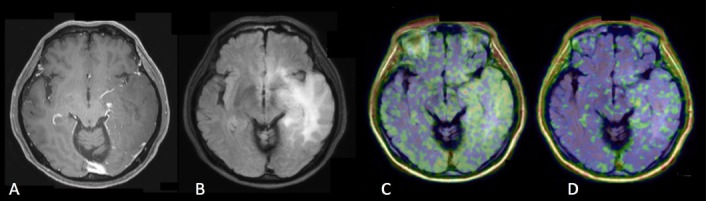
A: Gd contrast-enhanced T1-weighted image B: FLAIR (fluid-attenuated inversion recovery) image C: [^11^C]methionine positron emission tomography (MET-PET) D: anti- [^18^F]FACBC positron emission tomography (FACBC-PET) A 61-year-old man who had a diffuse irregular high signal lesion in the left frontal lobe in FLAIR image without enhancement in Gd T1-weighted image. The lesion was diagnosed with diffuse astrocytoma, grade II, IDH mutant. Slight uptake of MET and FACBC was detected in the lesion, but it was difficult to confirm the tumor border in both tracers.

**Figure 4. Case 3:**
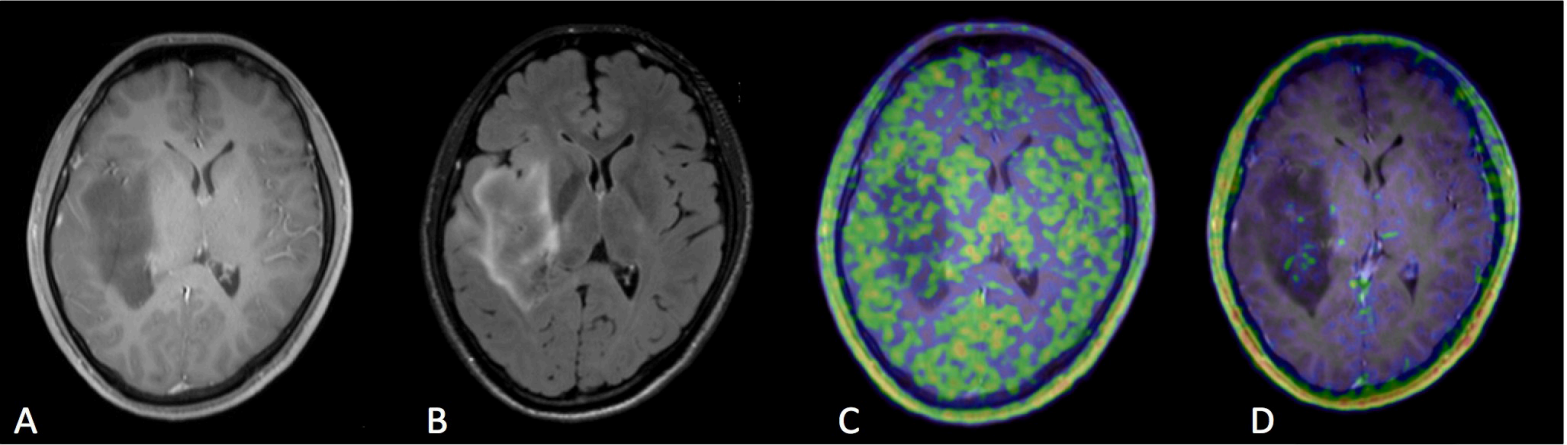
A: Gd contrast-enhanced T1-weighted image B: FLAIR (fluid-attenuated inversion recovery) image C: [^11^C]methionine positron emission tomography (MET-PET) D: anti- [^18^F]FACBC positron emission tomography (FACBC-PET) In a 21-year-old man, the lesion was confirmed as a diffuse low signal lesion without enhancement in Gd T1-weighted image and a slight high irregular lesion in FLAIR image on the left insula lobe. The lesion was diffuse astrocytoma, grade II, IDH mutant. MET-PET showed low uptake in the lesion as well as background. On the other hand, FACBC-PET showed low uptake in the lesion, but slight accumulation was found in small part of the tumor.

**Figure 4. Case 4:**
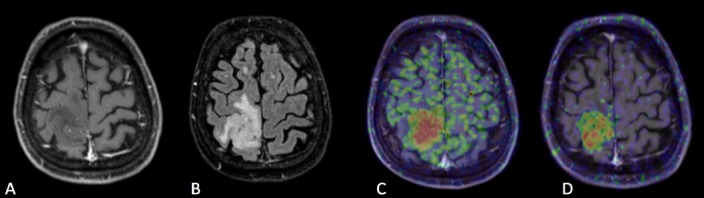
A: Gd contrast-enhanced T1-weighted image B: FLAIR (fluid-attenuated inversion recovery) image C: [^11^C]methionine positron emission tomography (MET-PET) D: anti- [^18^F]FACBC positron emission tomography (FACBC-PET) A 57-year-old man who had a small round-shaped mass without enhancement in the right frontal lobe in Gd T1-weighted image, which was diagnosed with oligodendroglioma, grade II, IDH mutant, 1p/19q co-deletion. This case showed 1p19q co-deletion. Both PET tracers were detected in a high signal lesion of FLAIR image, and it was easy to detect the border between tumor and cortex. FACBC-PET showed clearer contrast of abnormal lesion than MET-PET.

**Figure 4. Case 5:**
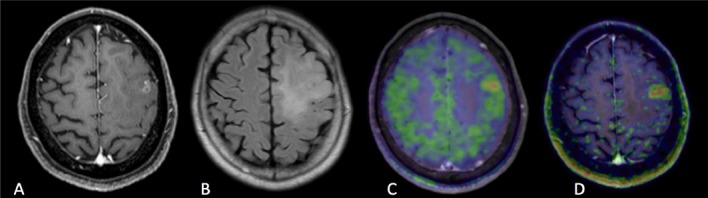
A: Gd contrast-enhanced T1-weighted image B: FLAIR (fluid-attenuated inversion recovery) image C: [^11^C]methionine positron emission tomography (MET-PET) D: anti- [^18^F]FACBC positron emission tomography (FACBC-PET) A 70-year-old lady had a slight irregular round-shaped mass in the left frontal lobe. Gd T1-weighted image showed a slight enhancement into the high signal area in FLAIR image. The lesion was diagnosed with anaplastic astrocytoma, IDH wild-type. Though high uptake of MET and FACBC was found in the tumor, FACBC detected more clearly the tumor border than MET. The slight high signal lesion in FLAIR was considered to be edema.

**Figure 4. Case 6:**
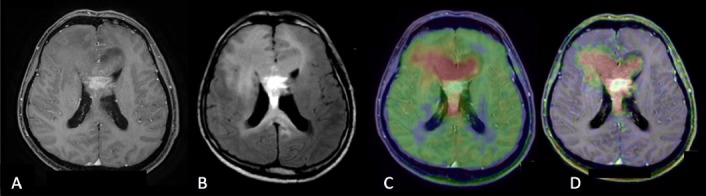
A: Gd contrast-enhanced T1-weighted image B: FLAIR (fluid-attenuated inversion recovery) image C: [^11^C]methionine positron emission tomography (MET-PET) D: anti- [^18^F]FACBC positron emission tomography (FACBC-PET) A 43-year-old man had well-enhanced irregular mass in the corpus callosum of the left frontal lobe in Gd T1-weighted image. The lesion invaded bilateral frontal lobes in FLAIR image. The diagnosis was glioblastoma, IDH mutant. Both MET and FACBC-PET showed high uptake in the tumor and wider area than enhancement lesion in Gd T1-weighted image and smaller than high signal lesion in FLAIR image. A clear margined tumor was depicted in FACBC-PET.

### Illustrated cases

Case 1, a 40-year-old lady, was diagnosed with Grade II, IDH mutant, diffuse astrocytoma (Figure 4; Case 1). Gadolinium-enhanced (Gd) T1-weighted imaging showed low signal round shape mass with no enhancement in the left frontal lobe, however, there was very small lesion with poor enhancement within the mass. FLAIR imaging showed a high signal lesion around the tumor. Both MET-PET and FACBC-PET demonstrated tracer uptake of the poor enhancement lesion, which was diagnosed as the main tumor. Within the FLAIR high lesion, any tumor cells could not be detected as the result of surgery and was diagnosed as edema. High uptake of MET was found in a small part of the tumor, while the slight uptake of FACBC was clearly detectable against a low background.

Case 2, a 61-year-old man, was diagnosed with Grade II, IDH mutant, diffuse astrocytoma (Figure 4; Case 2). FLAIR imaging showed a diffuse and irregular high signal lesion in the left temporal lobe, with no enhancement in the Gd T1-weighted image. Slight uptake of MET and FACBC was detected in the lesion, but it was difficult to confirm the tumor border with either tracer.

Case 3, a 21-year-old man, was also diagnosed with Grade II, IDH mutant, diffuse astrocytoma (Figure 4; Case 3). The lesion had a diffuse, low signal without enhancement in Gd T1-weighted imaging, and a slightly high, irregular lesion detected by FLAIR imaging on the left insula lobe. MET-PET showed low uptake in both the lesion and background. On the other hand, while FACBC-PET showed generally low uptake in the lesion, a slight accumulation was found in a small part of the tumor.

Case 4, a 57-year-old man, was diagnosed with Grade II, IDH mutant oligodendroglioma, featuring TERT mutation and 1p19q co-deletion (Figure 4; Case 4). Gd T1-weighted imaging revealed a small round mass without enhancement in the right frontal lobe. Both PET tracers were taken up by the high signal lesion revealed by FLAIR imaging, and the border between the tumor and cortex was easily detectable. FACBC-PET showed greater contrast of the abnormal lesion than MET-PET.

Case 5, a 70-year-old lady, was diagnosed with IDH wild-type, anaplastic astrocytoma (Figure 4; Case 5). Gd T1-weighted imaging showed slight enhancement of the irregular high signal mass observed by FLAIR. Though both PET approaches could detect the tumor in the left frontal lobe, FACBC-PET showed the high cellularity border more clearly than MET-PET. The slight high signal lesion in FLAIR demonstarted edema as the result of tumor resection.

Case 6, a 43-year-old man, was diagnosed with IDH mutant glioblastoma (Figure 4; Case 6). Gd T1-weighted imaging showed a well-enhanced irregular mass in the left corpus callosum; however, FLAIR imaging revealed that the high signal lesion was spread wider than observed by Gd. Both MET-PET and FACBC-PET could detect the tumor in the bilateral frontal lobe, which was larger than that observed by Gd imaging. Once again, FACBC-PET displayed the tumor border against normal cortex more clearly than MET-PET.

## Discussion

The present study indicated several differences between FACBC-PET and MET-PET regarding intracranial imaging. FACBC-PET demonstrated lower tracer uptake than MET-PET in normal brain tissue, in both gray and white matter, and similar uptake to MET in the glandular system (pituitary gland, lacrimal grand, choroid plexus, etc.), mucosa, and the bone marrow. Lower background uptake leads to greater lesion contrast and easier detection of tumors. Our data indicated that FACBC-PET performs better than MET-PET for visual assessment in glioma. The L/N_mean_ and L/N_max_ with FACBC-PET was higher than those with MET due to the low uptake of FACBC by normal brain (Figures 1 and 2). Moreover, FACBC-PET may contribute to more accurate delineation of tumor margins when stereotactic surgery, biopsy, or radiotherapy is considered for the treatment of malignant brain tumors. In terms of uptake values, the SUV_max_ of FACBC in glioma showed a weak, non-significant, linear relationship with that of MET.

MET-PET has been widely used for brain tumor imaging, and in glioma patients it has been extremely useful not only for detecting astrocytoma, but also for differentiating between benign and malignant astrocytoma. However, some reports indicated that MET SUV_max_ and L/N ratio are not significantly different between Grade II and Grade III gliomas and that low-grade oligo-type gliomas have relatively high MET uptake (1, 11), that is, MET-PET may not be useful for determining the histological grade of astrocytoma. In the present study, FACBC-PET demonstrated potential to distinguish between low-grade (Cases 1, 2, and 3) and high-grade astrocytomas (Cases 5 and 6) using SUVs and L/N ratio, but it is difficult to draw definitive conclusions regarding the utility of this technique for tumor grading given the small number of examined cases.

Consistent with a previous report (12), both MET and FACBC are readily taken up by oligodendrogliomas. Regarding the uptake mechanism of the amino acid tracer, oligodendrogliomas are assumed to have high cell density, endothelial hyperplasia, and high vascularization in tumor beds compared to astrocytomas, so many factors are thought to be involved.

Although the true mechanism is inconspicuous, it is possible that the 1p19q co-deletion may play a role. Both FACBC-PET and MET-PET tend to show high accumulation in gliomas with a high MIB-1 index (i.e., those with high rates of proliferation). In cases with TERT mutation (e.g., Cases 5 and 6), the accumulation of FACBC also tends to be high. It has been found that TERT mutation is a promising predictor of prognosis in patients with glioblastoma (13). It is necessary to study more cases to properly evaluate the relationship between FACBC uptake and TERT mutation.

Physiological uptake of MET is considered to occur via system L-amino acid transporters because MET is utilized by normal brain tissues as a substrate for protein synthesis, neurotransmitter synthesis, and energy production. In case of FACBC, *in vitro* studies on prostate cancer and glioma indicated that the alanine-serine-cysteine transporters (ASCT), especially ASCT2, and L-type amino acid transporters (LAT), especially LAT1, are involved in its uptake (14-16). This suggests that FACBC-PET may be particularly useful for the diagnosis of malignant brain tumors with high expression of system L- and system ASC-amino acid transporters. Nonetheless, unlike MET, the artificial amino acid FACBC is not able to enter normal metabolic pathways (17), which may explain its lower rates of accumulation in healthy tissues. Indeed, in this study, FACBC-PET displayed higher diagnostic accuracy than MET-PET, with high LN ratios and good contrast between the marginal edge of gliomas and surrounding tissues, which may result in lower false positives in FACBC-PET than MET-PET. Nevertheless, this observation needs to be confirmed in a larger cohort of patients.

## Conclusion

Overall, FACBC-PET provided better assessment for detecting gliomas than MET-PET. In terms of differential diagnosis between low- and high-grade astrocytoma, FACBC-PET was found to show the same diagnostic potential as MET-PET when taking into consideration SUVs and LN ratios. Limitation of this study was its small sample size. To confirm our observations, further investigations on other types of brain tumor, such as metastatic brain tumors and central nervous system lymphoma, are required.
